# Association between the C-reactive protein/albumin ratio and prognosis in patients with oral squamous cell carcinoma

**DOI:** 10.1038/s41598-021-83362-2

**Published:** 2021-03-08

**Authors:** Kenji Yamagata, Satoshi Fukuzawa, Naomi Ishibashi-Kanno, Fumihiko Uchida, Hiroki Bukawa

**Affiliations:** Department of Oral and Maxillofacial Surgery, Institute of Clinical Medicine, Faculty of Medicine, University of Tsukuba, 1-1-1 Tennodai, Tsukuba, Ibaraki 305-8575 Japan

**Keywords:** Head and neck cancer, Oral cancer

## Abstract

The systemic inflammatory response is known to be associated with poor outcomes in patients with various types of cancer. The C-reactive protein (CRP)/albumin (Alb) ratio (CAR) has been reported as a novel inflammation-based prognostic marker. We have evaluated the prognostic value of inflammatory markers for patients with oral squamous cell carcinoma (OSCC). The study population included 205 patients treated with OSCC between 2013 and 2018. The primary predictor variable was the inflammatory markers. The primary outcome variable was overall survival (OS). Univariate and multivariate analyses were performed using a Cox proportional hazards model to identify independent prognostic factors. The CAR had the highest area under the curve (AUC) values compared with other markers in the receiver operating characteristic (ROC) curve analysis. The cutoff value for CAR was 0.032 (AUC 0.693, *P* < 0.001). There was a significant difference in OS when patients were stratified according to CAR, with 79.1% for CAR < 0.032 and 35% for CAR ≥ 0.032 (*P* < 0.001). Cox multivariate analysis identified independent predictive factors for OS: age (hazard ratio [HR] 2.155, 95% confidence interval [CI] 1.262–3.682; *P* = 0.005), stage (HR 3.031, 95% CI 1.576–5.827; *P* = 0.001), and CAR (HR 2.859, 95% CI 1.667–4.904; *P* < 0.001). CAR (≥ 0.032 vs. < 0.032) is a good prognostic marker in patients with OSCC in terms of age and stage.

## Introduction

Host and tumor factors interact, and these interactions can either accelerate tumor progression or regression. The systemic inflammatory response is associated with poor outcomes in patients diagnosed with various malignancies^1^. Several common inflammation-based markers, including the neutrophil/lymphocyte ratio (NLR), platelet/lymphocyte ratio (PLR), lymphocyte/monocyte ratio (LMR), systemic inflammation response index (SIRI), and systemic immune-inflammation index (SII), have been reported previously^2–5^.

The C-reactive protein (CRP)/albumin (Alb) ratio (CAR) has been identified as a novel inflammation-based prognostic marker in several cancers, including esophageal cancer, lung cancer, hypopharyngeal and laryngeal cancer, and nasopharyngeal cancer^6–9^. CAR is an independent marker of inflammation in various cancers and a more accurate prognostic marker than other markers, such as the modified Glasgow prognostic score (mGPS), NLR, and PLR^10–12^. These inflammation-based prognostic scores can be easily and routinely measured and serve as a valuable prognostic parameter.

To the best of our knowledge, only two reports examining the prognostic value of CAR in oral squamous cell carcinoma (OSCC) have been published^13,14^. Therefore, the present study aimed to evaluate and compare the prognostic value of inflammatory markers, including the NLR, SIRI, SII, LMR, and the novel prognostic factor, CAR, in patients with OSCC.

## Results

### Optimal cutoff values of the inflammation-based prognostic scores

**Figure 1:**
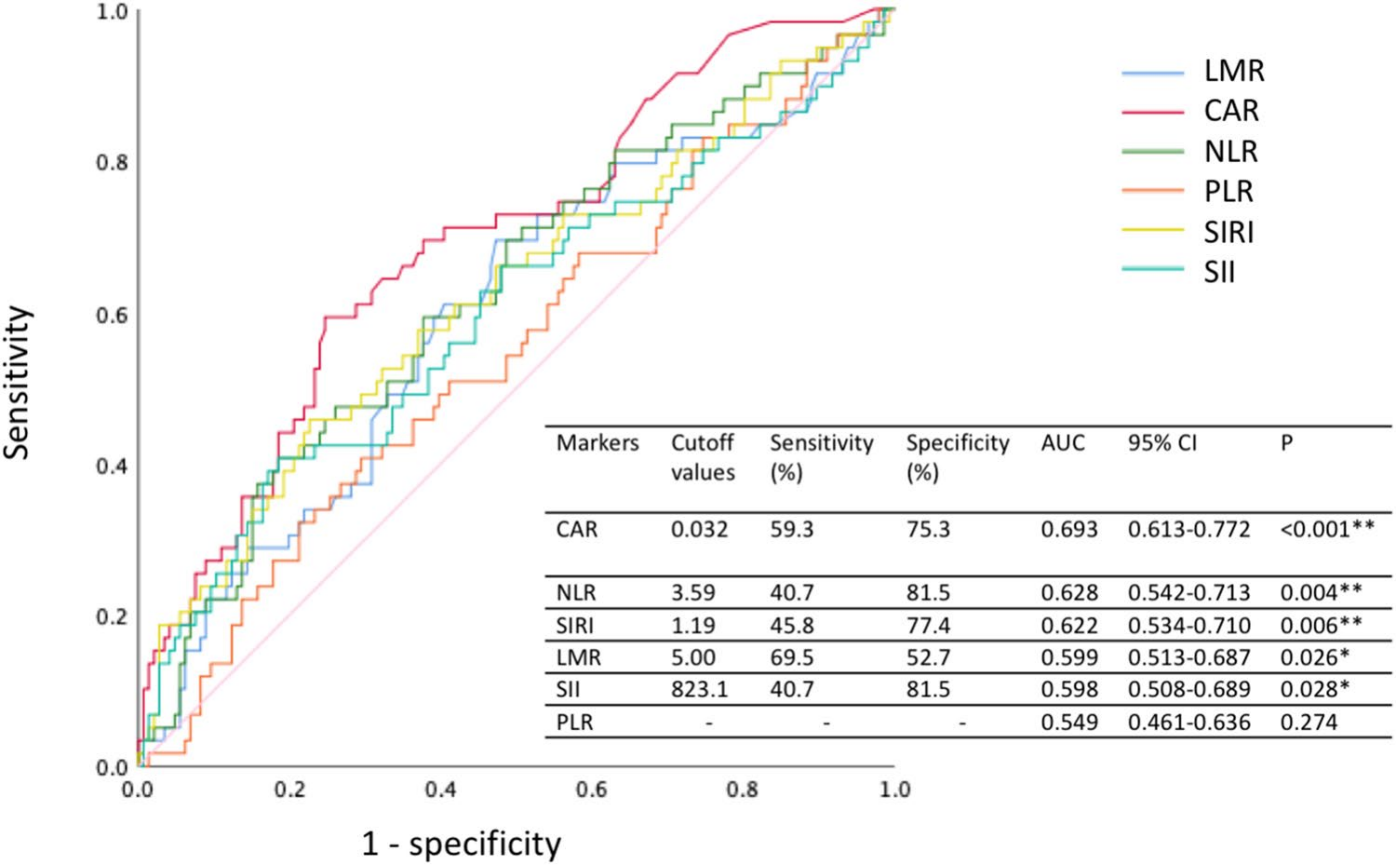
Receiver operating characteristic (ROC) curves of the inflammatory markers analyzed as predictors of the overall survival (OS).

The cutoff values for predicting OS were calculated by the receiver operating characteristic (ROC) curve analysis, which determined that the optimal cutoff values for NLR, SIRI, SII, LMR, and CAR were 3.59 (area under the curve [AUC], 0.628; sensitivity, 40.7%; specificity, 81.5%; *P* = 0.004), 1.19 (AUC, 0.622; sensitivity, 45.8%; specificity, 77.4%; *P* = 0.006), 823.1 (AUC, 0.598; sensitivity, 40.7%; specificity, 81.5%; *P* = 0.028), 5.00 (AUC, 0.599; sensitivity, 69.5%; specificity, 52.7%; *P* = 0.026), and 0.032 (AUC, 0.693; sensitivity, 59.3%; specificity, 75.3%; *P* < 0.001), respectively. CAR had the highest AUC value (Fig. 1).

### Association between patient characteristics and the CAR cutoff value

Based on the CAR cutoff value, the patients were subdivided into two groups: 134 patients presented with a low CAR (< 0.032) and 71 had a high CAR (≧ 0.032). The median age was 71.3 (range: 31.1–93.0) years. Age differed significantly between the two groups (*P* = 0.007). The patients included 123 men and 82 women with a median pretreatment BMI of 22.49 (range: 13.51–34.21) kg/m^2^. The most common primary tumor sites included the tongue (n = 76), mandibular gingiva (n = 65), and buccal mucosa (n = 22). There was a significant difference in the primary site between the two groups (*P* = 0.003). Although there was no significant difference in the mandibular gingiva between the two groups, a significant difference occurred in the tongue vs. others between the two groups (*P* = 0.002). The TNM classification and stage details are shown in Table 1. There were significant differences in TN classification and stage between the low and high CAR groups (*P* < 0.01). Management involved only surgery in 89 patients with a low CAR and 27 patients with a high CAR, surgery with or without radiotherapy or chemoradiotherapy in 36 patients with a low CAR and 15 patients with a high CAR, and only radiotherapy in 9 patients with a low CAR and 29 patients with a high CAR. There was a significant difference in the management between the three groups (*P* < 0.001). The other primary predictor variables that were analyzed in addition to CAR included NLR, SIRI, SII, and LMR. These variables were dichotomized according to predetermined cutoff values, and similar to CAR, all demonstrated significant differences between the two groups (*P* < 0.001). The results of the other patient-related characteristics/variables are presented in Table 1.

**Table 1:** Clinical characteristics of the patients included in the study dichotomized according to the cutoff value of CAR.

Variable		Total No. of patients	CAR < 0.032 No. of patients (%) n = 134	CAR ≧ 0.032 No. of patients (%) n = 71	*P* value
Age (years)					0.007**
Median (range)	71.3 (31.1–93.0)		71.0 (31.1–93.0)	73.2 (49.3–92.6)	
BMI (kg/m^2^)					0.211
Median (range)	22.49 (13.51–34.21)		22.65 (13.51–32.54)	22.14 (15.84–34.21)	
Gender	Male	123	80 (65.0)	43 (35.0)	0.905
	Female	82	54 (65.9)	28 (34.1)	
Tabaco consumption	Present	27	16 (59.3)	11 (40.7)	0.754
	Pre	31	20 (64.5)	11 (35.5)	
	Never	147	98 (66.7)	49 (33.3)	
Alcohol consumption	Present	65	44 (67.7)	21 (32.3)	0.633
	None	140	90 (64.3)	50 (35.7)	
Primary site	Tongue	76	60 (78.9)	16 (21.1)	0.003**
	Lower gingiva	65	40 (61.5)	25 (38.5)	
	Buccal mucosa	22	14 (63.6)	8 (36.4)	
	Upper gingiva	19	9 (47.4)	10 (52.6)	
	Floor of the mouth	13	7 (53.8)	6 (46.2)	
	Others	10	4 (40.0)	6 (60.0)	
T classification	T1	40	37 (92.5)	3 (7.5)	< 0.001**
	T2	62	46 (74.2)	16 (25.8)	
	T3	35	24 (68.6)	11 (31.4)	
	T4a	56	24 (42.9)	32 (57.1)	
	T4b	12	3 (25.0)	9 (75.0)	
N classification	N0	132	94 (71.2)	38 (28.8)	0.003**
	N1	25	18 (72.0)	7 (28.0)	
	N2a	1	1 (100)	0 (0)	
	N2b	35	19 (54.3)	16 (45.7)	
	N2c	10	2 (20.0)	8 (80.0)	
	N3b	2	0 (0)	2 (100)	
M classification	M0	204	134 (65.7)	70 (34.3)	0.346
	M1	1	0 (0)	1 (100)	
Stage	I	38	35 (92.1)	3 (7.9)	< 0.001**
	II	52	39 (75.0)	13 (25.0)	
	III	28	20 (71.4)	8 (28.6)	
	IVA	73	37 (50.7)	36 (49.3)	
	IVB	13	3 (23.1)	10 (76.9)	
	IVC	1	0 (0)	1 (100)	
Histological grade	Well	105	71 (67.6)	34 (32.4)	0.225
	Moderate	80	53 (66.3)	27 (33.7)	
	Poor	11	4 (36.4)	7 (63.6)	
	Others	9	6 (66.7)	3 (33.3)	
NLR	≥ 3.59	51	21 (41.2)	30 (58.8)	< 0.001**
	< 3.59	154	113 (73.4)	41 (26.6)	
SIRI	≥ 1.19	59	24 (40.7)	35 (59.3)	< 0.001**
	< 1.19	146	110 (75.3)	36 (24.7)	
SII	≥ 823.1	51	20 (39.2)	31 (60.8)	< 0.001**
	< 823.1	154	114 (74.0)	40 (26.0)	
LMR	≥ 5.0	95	76 (80.0)	19 (20.0)	< 0.001**
	< 5.0	110	58 (52.7)	52 (47.3)	
Management	Surgery	116	89 (76.7)	27 (23.3)	< 0.001**
	Surgery + radiotherapy or chemoradiotherapy	51	36 (70.6)	15 (29.4)	
	Radiotherapy	38	9 (23.7)	29 (76.3)	
Local recurrence	Present	39	15 (38.5)	24 (61.5)	< 0.001**
	None	166	119 (71.7)	47 (28.3)	
Neck recurrence	Present	15	9 (60.0)	6 (40.0)	0.650
	None	190	125 (65.8)	65 (34.2)	
Distant metastasis	Present	20	13 (65.0)	7 (35.0)	0.971
	None	185	121 (65.4)	64 (34.6)	

*P < 0.05 Statistically significant difference.

**P < 0.01 Statistically significant difference.

NLR, neutrophil/lymphocyte ratio; SIRI, systemic Inflammation Response Index; LMR, lymphocyte/ monocyte ratio; SII, systemic immune-inflammation index; CAR, C-reactive protein/albumin ratio.

Of the 205 patients with OSCC, 146 (71.2%) survived during the follow-up period; among these, OSCC recurred locally in 39 patients (19.0%), spread regionally in 15 (7.3%), and metastasized distantly in 20 (9.8%). Of the 59 patients (28.8%) who died during the follow-up period, 52 died with OSCC and 7 died without any OSCC recurrence or metastasis. Local recurrence occurred in 15 (38.5%) patients with a low CAR and 24 (61.5%) with a high CAR, and there was a significant difference between the two groups (*P* < 0.001) (Table 1).

### Association between clinical factors and OS

**Figure 2:**
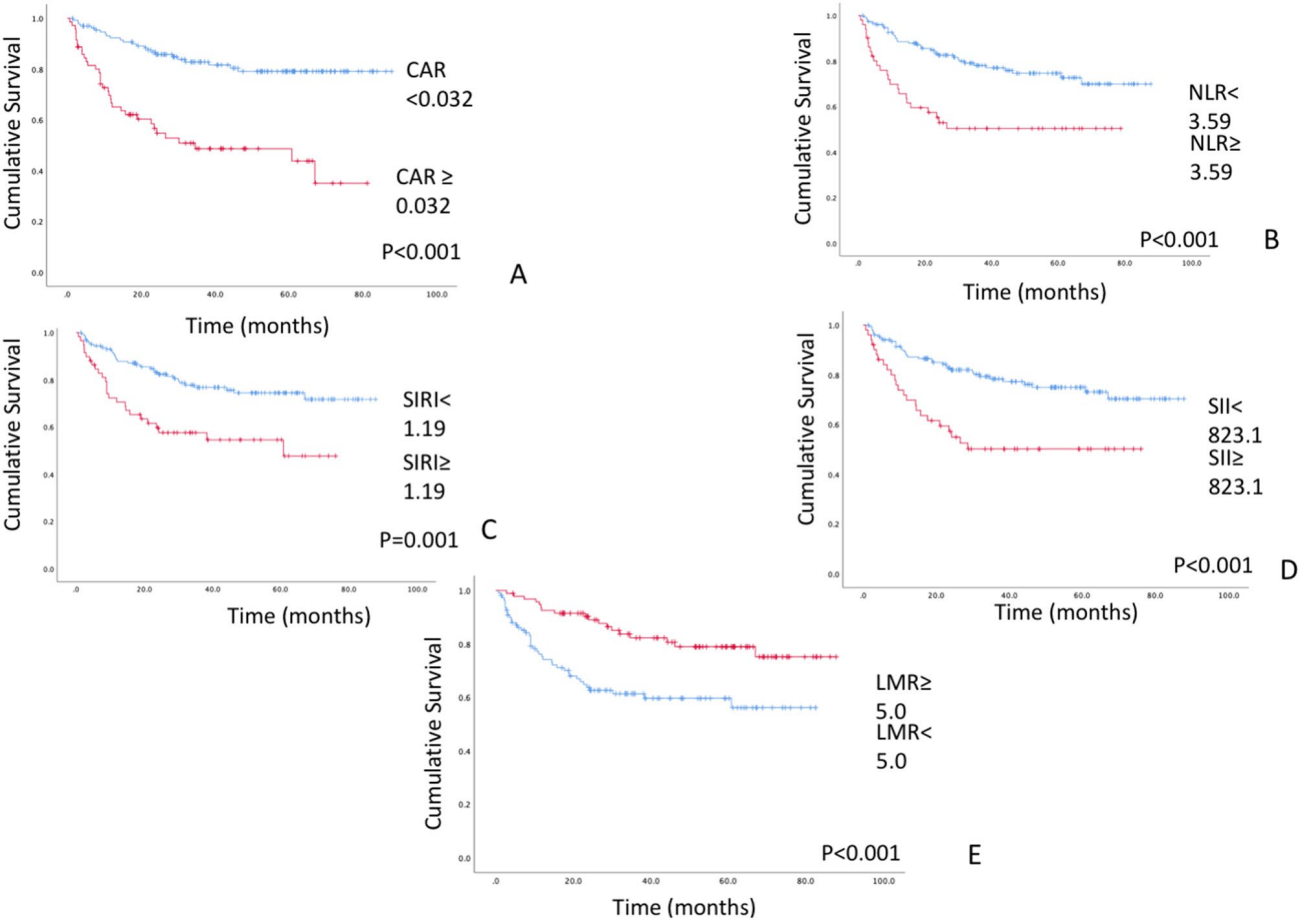
(**A**) Kaplan–Meier survival curve according to CAR. Patients were stratified according to the cutoff value of CAR (0.032). The OS rate was 79.1% for patients with a low CAR (< 0.032) and 35% for a high CAR (≥ 0.032) (*P* < 0.001). (**B**)–(**E**) Kaplan–Meier survival curves according to NLR, SIRI, SII, and LMR. Significant differences in OS were noted when the patient cohort was subdivided into two groups according to the cutoff value of each inflammatory marker (NLR < 3.59, 70.0% vs. NLR ≥ 3.59, 50.4%, *P* < 0.001; SIRI < 1.19, 71.7% vs. SIRI ≥ 1.19, 47.7%, *P* = 0.001; SII < 823.1, 70.3% vs. SII ≥ 823.1, 50.1%, *P* < 0.001; and LMR ≥ 5.0, 75.2% vs. LMR < 5.0, 56.1%, *P* < 0.001). A less favorable prognosis was associated with a higher NLR, SIRI, and SII values and a lower LMR value.

The median follow-up period of the survivors was 45.8 (1.4–87.8) months. The associations between the study variables and OS are presented in Table 2. There were significant differences in OS when patients were stratified according to stage classification (OS rates: stage I, 88.0%; stage II, 71.5%; stage III, 77.4%; stage IVA, 49.4%; stage IVB, 44.9%; and stage IVC, 0%; *P* = 0.001; Supplementary Fig. 1). There were significant differences in OS with age and TNM classification (*P* < 0.01). In the primary site, there was a significant difference in OS when patients stratified the tongue and others (OS rates: tongue 79.9% vs. others 55.8%; *P* = 0.007). Likewise, significant differences in the primary outcome variable (OS) were obtained when patients were stratified according to the primary predictor variable (CAR), with an OS rate of 79.1% for CAR < 0.032 and 35% for CAR ≥ 0.032 (*P* < 0.001; Fig. 2A). There were significant differences in the OS curve when patients were divided by NLR < 3.59 (70.0%) vs. NLR ≥ 3.59 (50.4%; *P* < 0.001), SIRI < 1.19 (71.7%) vs. SIRI ≥ 1.19 (47.7%; *P* = 0.001), SII < 823.1 (70.3%) vs. SII ≥ 823.1 (50.1%; *P* < 0.001), and LMR ≥ 5.0 (75.2%) vs. LMR < 5.0 (56.1%; *P* < 0.001) (Fig. 2B–E).

**Table 2:** Characteristics of OSCC patients in relation to cumulative survival.

Variables		No. of patients (%)	OS (%)	*P*^†^
Age	≥ 71.3	103 (50.2)	56.9	0.006**
(years)	< 71.3	102 (49.8)	72.8	
Gender	Male	123 (60.0)	68.6	0.501
	Female	82 (40.0)	60.2	
Tabaco consumption	Present	27 (13.2)	48.9	0.444
	Pre	31 (15.1)	77.2	
	Never	147 (71.7)	66.8	
Alcohol consumption	Present	65 (31.7)	69.9	0.348
	None	140 (68.3)	62.3	
Primary site	Tongue	76 (37.1)	79.9	0.069
	Lower gingiva	65 (31.7)	55.9	
	Buccal mucosa	22 (10.7)	62.2	
	Upper gingiva	19 (9.3)	61.5	
	Floor of the mouth	13 (6.3)	39.5	
	Others	10 (4.9)	60.0	
Stage	I	38 (18.5)	88.0	< 0.001**
	II	52 (25.4)	71.5	
	III	28 (13.7)	77.4	
	IV A	73 (35.6)	49.4	
	IV B	13 (6.3)	44.9	
	IV C	1 (0.5)	0	
T classification	T1	40 (19.5)	89.0	< 0.001**
	T2	62 (30.2)	67.1	
	T3	35 (17.1)	76.0	
	T4a	56 (27.3)	43.8	
	T4b	12 (5.9)	40.0	
N classification	N0	132 (64.4)	71.5	0.002**
	N1	25 (12.2)	62.3	
	N2a	1 (0.5)	0	
	N2b	35 (17.1)	56.8	
	N2c	10 (4.9)	19.0	
	N3b	2 (1.0)	50.0	
M classification	M0	204 (99.5)	65.3	< 0.001**
	M1	1 (0.5)	0	
Histological grade	Well	105 (51.2)	73.7	0.106
	Moderate	80 (39.0)	59.7	
	Poor	11 (5.4)	25.5	
	Others	9 (4.4)	66.7	
NLR	≥ 3.59	51 (24.9)	50.4	< 0.001**
	< 3.59	154 (75.1)	70.0	
SIRI	≥ 1.19	59 (28.8)	47.7	0.001**
	< 1.19	146 (71.2)	71.7	
SII	≥ 823.1	51 (24.9)	50.1	< 0.001**
	< 823.1	154 (75.1)	70.3	
LMR	≥ 5.0	95 (46.3)	75.2	< 0.001**
	< 5.0	110 (53.7)	56.1	
CAR	≥ 0.032	71 (34.6)	35.0	< 0.001**
	< 0.032	134 (65.4)	79.1	
CRP	≥ 0.105	80 (39.0)	40.6	< 0.001**
(mg/dL)	< 0.105	125 (61.0)	78.3	
Alb	≥ 4.15	114 (55.6)	76.0	< 0.001**
(g/dL)	< 4.15	91 (44.4)	51.5	
Management	Surgery	116 (56.6)	92.1	< 0.001**
	Surgery + radiotherapy or chemoradiotherapy	51 (24.9)	51.3	
	Radiotherapy	38 (18.5)	15.5	
Local recurrence	Present	39 (19.0)	10.0	< 0.001**
	None	166 (81.0)	79.5	
Neck recurrence	Present	15 (7.3)	6.7	< 0.001**
	None	190 (92.7)	71.3	
Distant metastasis	Present	20 (9.8)	14.3	< 0.001**
	None	185 (90.2)	70.8	

^†^By log-rank test.

*P < 0.05 Statistically significant difference, **P < 0.01 Statistically significant difference.

OS, overall survival; NLR, neutrophil/lymphocyte ratio; SIRI, systemic inflammation response index; LMR, lymphocyte/monocyte ratio; SII, systemic immune-inflammation index; CAR, C-reactive protein albumin/albumin ratio.

In the Kaplan–Meier analysis and the cumulative survival curves of patients stratified into quartiles on the basis of the CAR value range, there were significant differences in OS when patients were stratified into quartiles according to the CAR range (OS rates: CAR < 0.0073, 86.6%; 0.0073 ≤ CAR < 0.0167, 74.9%; 0.0167 ≤ CAR < 0.0569, 50.3%; 0.0569 ≤ CAR, 47.0%; *P* < 0.001).

### Subgroup analysis with management of the surgery group

In 167 patients (81.5%) in the surgery cohort, univariate analyses showed that OS was significantly associated with CAR (≥ 0.032 vs. < 0.032), with a hazard ratio [HR] of 2.262 and 95% confidence interval [CI] of 1.087–4.705 (*P* = 0.029). Significant associations of OS with NLR (≥ 3.59 vs. < 3.59; HR 2.181, 95% CI 1.021–4.662;* P* = 0.044) and SII (≥ 823.1vs. < 823.1; HR 2.623, 95% CI 1.262–5.449; *P* = 0.010) were also identified. As postoperative risk factors, extra nodal extension (ENE) and positive LN counts (< 2 or ≥ 2) were analyzed, showing that OS was significantly associated with ENE (present vs. absent; HR 9.360, 95% CI 3.724–23.526; *P* < 0.001) and LN counts (< 2 or ≥ 2; HR 3.043, 95% CI 1.423–6.507; *P* < 0.004) (Table 3).

**Table 3:** Subgroup analysis with univariate cox regression analyses for OS in the surgery group.

Variables	Univariate analysis	
	HR (95% CI)	*P* values ^†^
**Gender**		
Male vs. Female	0.862 (0.419–1.775)	0.687
**Age**		
≥ 71.3 vs. < 71.3	1.696 (0.827–3.480)	0.150
**Stage**		
I, II vs. III, IV	1.861 (0.885–3.914)	0.101
**T stage**		
T1,2 vs. 3,4	1.567 (0.764–3.211)	0.220
**N classification**		
N0, 1 vs. N2, 3	2.039 (0.933–4.457)	0.074
**NLR**		
≥ 3.59 vs. < 3.59	2.181 (1.021–4.662)	0.044*
**SIRI**		
≥ 1.19 vs. < 1.19	1.981 (0.941–4.168)	0.072
**SII**		
≥ 823.1vs. < 823.1	2.623 (1.262–5.449)	0.010*
**LMR**		
≥ 5.0 vs. < 5.0	0.567 (0.275–1.168)	0.124
**CAR**		
≥ 0.032 vs. < 0.032	2.262 (1.087–4.705)	0.029*
**Pathological ENE**		
Present or absent	9.360 (3.724–23.526)	< 0.001**
**Positive LN counts**		
≥ 2 vs. < 2	3.043 (1.423–6.507)	0.004**

^†^By Cox proportional hazards regression.

*P < 0.05 Statistically significant difference, **P < 0.01 Statistically significant difference.

NLR, neutrophil lymphocyte ratio; SIRI, systemic inflammation response index; LMR, lymphocyte monocyte ratio; SII, systemic immune-inflammation index; CAR, C-reactive protein/albumin ratio; ENE, Extra nodal extension; LN, Lymph node.

### Subgroup analysis according to stage and CAR

**Figure 3:**
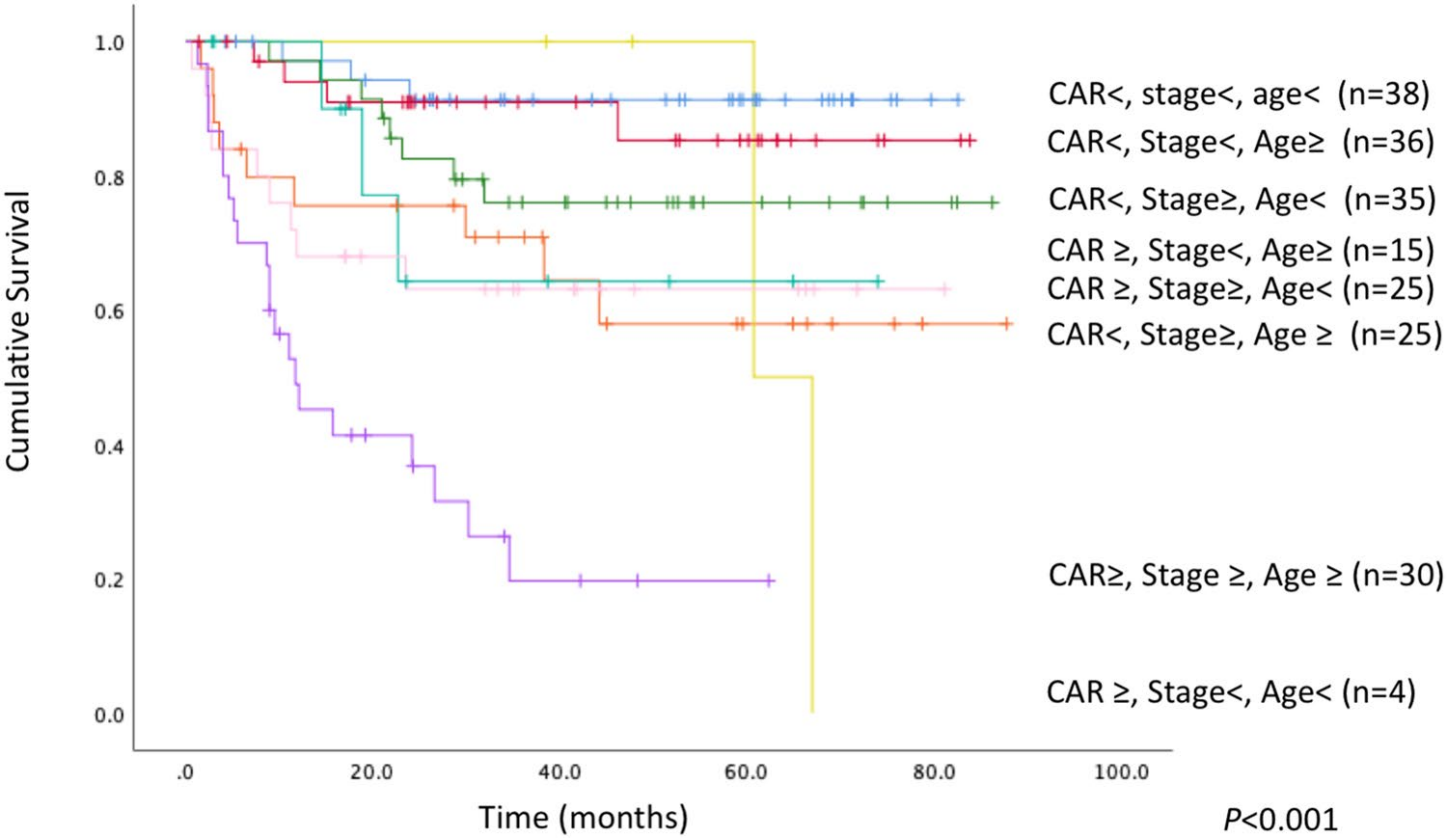
Kaplan–Meier survival curve according to a combination of CAR (< 0.032 or ≥ 0.032), stage (I, II or III, IV), and age (< 71.3 or ≥ 71.3). There were significant differences in OS when the patient cohort was stratified according to a combination of CAR and stage (OS rates: CAR <, stage <, age <, 91.3%; CAR <, stage <, age ≥, 85.3%; CAR <, stage ≥, age <, 76.0%; CAR ≥, stage <, age ≥, 64.3%; CAR ≥, stage ≥, age <, 63.1%; CAR <, stage ≥, age ≥ 58.0%; CAR ≥, stage ≥, age ≥, 19.7%; CAR ≥, stage <, age <, 0%; *P* < 0.001).

A subgroup analysis was performed to evaluate the prognostic value of CAR when patients were stratified by stage and age. The OS results obtained for patients when grouped according to both CAR (< 0.032 or ≥ 0.032), stage (I, II or III, IV), and age (≥ 71.3 vs. < 71.3) are depicted in Fig. 3. There were significant differences in OS when patients were stratified by a combination of CAR, stage, and age (OS rates: CAR <, stage <, age <, 91.3%; CAR <, stage <, age ≥, 85.3%; CAR <, stage ≥, age <, 76.0%; CAR ≥, stage <, age ≥, 64.3%; CAR ≥, stage ≥, age <, 63.1%; CAR <, stage ≥, age ≥ 58.0%; CAR ≥, stage ≥, age ≥, 19.7%; CAR ≥, stage <, age <, 0%; *P* < 0.001). The combination of CAR <, stage <, and age < had the most favorable OS rate, while CAR ≥, stage ≥, age ≥, and CAR ≥, stage <, and age < had the least favorable prognosis.

### Cox multivariate regression analysis and logistic multivariate analysis

Univariate analyses showed that OS was significantly associated with CAR (≥ 0.032 vs. < 0.032), with an HR of 3.839 and 95% CI of 2.275–6.476 (*P* < 0.001). Significant associations between OS and age (≥ 71.3 vs. < 71.3; HR 2.077; 95% CI 1.224–3.527; *P* = 0.007), T classification (T1, 2 vs. T3, 4; HR 3.160; 95% CI 1.779–5.613; *P* < 0.001), N classification (N0, 1 vs. N2, 3; HR 2.285, 95% CI 1.347–3.878; *P* = 0.002), stage (I, II vs. III, IV; HR 3.617; 95% CI 1.918–6.824; *P* < 0.001), NLR (≥ 3.59 vs. < 3.59; HR 2.681; 95% CI 1.593–4.512;* P* < 0.001), SIRI (≥ 1.19 vs. < 1.19; HR 2.423; 95% CI 1.447–4.059; *P* = 0.001), SII (≥ 823.1vs. < 823.1; HR 2.578; 95% CI 1.532–4.340; *P* < 0.001), and LMR (≥ 5.0 vs. < 5.0; HR 0.380; 95% CI 0.218–0.663; *P* = 0.001) were also identified (Table 4).

**Table 4:** Univariate and multivariate cox regression analyses for OS in the primary cohort.

Variables	Univariate analysis		Multivariate analysis	
	HR (95% CI)	*P* values^†^	HR (95% CI)	*P* values^†^
**Gender**				
Male vs. Female	0.839 (0.502–1.402)	0.502	–	–
**Age**				
< 71.3vs. ≥ 71.3	2.077 (1.224–3.527)	0.007**	2.155 (1.262–3.682)	0.005**
**T classification**				
T1, 2 vs. T3, 4	3.160 (1.779–5.613)	< 0.001**		
**N classification**				
N0, 1 vs. N2, 3	2.285 (1.347–3.878)	0.002**		
**Stage**				
I, II vs. III, IV	3.617 (1.918–6.824)	< 0.001**	3.031 (1.576–5.827)	0.001**
**NLR**				
< 3.59 vs. ≥ 3.59	2.681 (1.593–4.512)	< 0.001**	–	–
**SIRI**				
< 1.19 vs. ≥ 1.19	2.423 (1.447–4.059)	0.001**	–	–
**SII**				
< 823.1vs. ≥ 823.1	2.578 (1.532–4.340)	< 0.001**	–	–
**LMR**				
< 5.0 vs. ≥ 5.0	0.380 (0.218–0.663)	0.001**	–	–
**CAR**				
< 0.032 vs. ≥ 0.032	3.839 (2.275–6.476)	< 0.001**	2.859 (1.667–4.904)	< 0.001**

^†^By Cox proportional hazards regression.

*P < 0.05 Statistically significant difference, **P < 0.01 Statistically significant difference.

NLR, neutrophil lymphocyte ratio; SIRI, systemic inflammation response index; LMR, lymphocyte monocyte ratio; SII, systemic immune-inflammation index; CAR, C-reactive protein/albumin ratio.

Logistic multivariate analysis of the parameters with stepwise forward selection method identified three independent predictive factors for survival: age (≥ 71.3 vs. < 71.3) (odds ratio [OR], 0.466; 95% CI, 0.235 to 0.924; *P* = 0.029), stage (I, II vs. III, IV) (OR, 0.269; 95% CI, 0.125 to 0.578; *P* = 0.001), and CAR (OR, 0.328; 95% CI, 0.166 to 0.648; *P* = 0.001); details are shown in Supplementary Table 1. In the Cox multivariate analysis, the parameters with significant variables by stepwise forward selection identified three independent predictive factors for OS: age (HR 2.155, 95% CI 1.262–3.682; *P* = 0.005), stage (HR 3.031, 95% CI 1.576–5.827; *P* = 0.001), and CAR (HR 2.859, 95% CI 1.667–4.904; *P* < 0.001). These results indicate that, of the variables analyzed in this study, age (≥ 71.3 vs. < 71.3), stage (I, II vs. III, IV), and CAR (≥ 0.032 vs. < 0.032) were more useful markers for predicting prognosis (Table 4).

## Discussion

The systemic inflammatory response influences carcinogenesis and cell proliferation, tumor cell migration, invasion, metastasis, cell survival, angiogenesis^15,16^. Kinoshita et al. demonstrated that CAR can serve as a novel inflammation-based prognostic score to predict survival in hepatocellular carcinoma^12^. The inflammatory marker, CRP, and nutritional marker, serum Alb, are widely used clinically and inexpensive to evaluate^17,18^. The CAR value provides an indication of the serum CRP concentrations relative to the serum concentration of Alb. A high CRP score may indicate an elevated serum CRP concentration in conjunction with hypoalbuminemia, an elevated CRP concentration relative to normal Alb levels, or normal CRP concentrations relative to a depressed Alb concentration. However, it has been reported that CAR may prove more useful in assessing disease status and predicting the long-term outcomes of malignancy^19^.

Secretion of pro-inflammatory cytokines, particularly interleukin 1 (IL-1) and 6 (IL-6), and chemokines, such as NF-κB, by tumor cells and tumor-infiltrating lymphocytes stimulates the immune and hematopoietic systems and upregulates the production of CRP, neutrophils, lymphocytes, platelets, and so on^20^. Serum CRP is produced mainly by hepatocytes, with production regulated by proinflammatory cytokines, especially IL-6. Therefore, CRP levels are indicative of tumor activity^19^. In the present study, a significant difference in OS between patients with high (≥ 0.105) and low CRP (< 0.105) levels was identified, with high CRP levels associated with a poorer prognosis. This result is consistent with the findings of previous reports.

Serum Alb is used as an indicator of nutritional status. Low levels are associated with poor survival outcomes in various cancers, including head and neck cancer (HANC)^17–19,21^. In the present study, a significant difference in OS between high (≥ 4.15) and low Alb (< 4.15) concentrations was noted, with hypoalbuminemia related to a significantly poor prognosis. Crumley et al. reported on the association between CRP and Alb and concluded that the relationship between hypoalbuminemia and poor survival was secondary to that of the systemic inflammatory response^21^. Similarly, according to Liu et al., prognosis was not significantly associated with CAR and preoperative BMI. In terms of CAR, the systemic inflammatory response exerted a more potent prognostic effect than nutritional status^22^. In the present patient cohort, there was no association between CAR and preoperative BMI, which corresponded with the suggestion that hypoalbuminemia and poor survival are secondary to the systemic inflammatory response.

Increased NLR has been found to be significantly associated with poor OS in patients with laryngeal cancer^23^. Rassouli et al. demonstrated that elevated PLR was correlated with higher mortality in HANC, suggesting that NLR and PLR as combined cellular components of systemic inflammation have a potential value for predicting cancer-specific survival in HANC^24^. In the present study, there were significant differences in OS between the two patient groups divided according to the cutoff values of NLR, SIRI, SII, LMR, and CAR. Moreover, the univariate Cox regression analysis showed significant differences in NLR, SIRI, SII, LMR, and CAR. However, it has been reported that CAR is superior to other inflammation-based prognostic scores, including NLR and PLR because CAR was found to have a higher AUC value than that obtained for the other markers^1,22^. Similarly, in the present study, the AUC value calculated for CAR on ROC curve analysis was found to be the highest. Moreover, on multivariate analysis, CAR, age, and stage were selected as independent predictors of OS. Therefore, CAR is currently recognized as the most useful prognostic marker. Furthermore, on Kaplan–Meier analysis, significant differences in OS were observed between groups when patients were stratified into quartiles, according to the CAR value range.

Table 5 summarizes the previous studies that have reported on the prognostic utility of CAR in various malignancies^1,5,8,10,12–14,19,20,22,25,26^. The cutoff values of CAR for the different cancers ranged from 0.023 to 0.525. Of the 13 studies, 5 (38.4%) were concerned with esophageal cancers, while only 3 (23.1%), including the present paper, related to OSCC. In HANC, excluding OSCC and including meta-analyses, three articles elucidating the prognostic value of CAR in nasopharyngeal cancer^9,25,27^ and one in hypopharyngeal cancer have been published^8^. Although the present study also demonstrated that CAR may be a more sensitive prognostic predictor in OSCC, the optimal predictive cutoff value differed from previously reported. Instead, a wide range of cutoff values were observed across the studies. This suggests that the significant CAR cutoff value may differ with the cancer type, pathological type, clinical stage, and type of treatment, and further research will be required to confirm this suspicion. In the previous two OSCC reports, the cutoff value of Wang et al. is 0.525, which is the highest of all CAR reports^13^. This value may be due to the different methods used to determine the cutoff value by Cutoff Finder analysis, the difference in the population of patients who had undergone surgery, and a short period of observation within 50 months. In contrast, Park et al. reported a cutoff value of 0.085^14^. Although this cutoff value is close to our cutoff value, the subjects were only 40 patients. A multiple center and large sample size analysis will be desired to determine the optimal cutoff value in patients with OSCC. In addition, there was a significant difference in the primary site of the tongue vs. others between CAR (≥ 0.032 vs. < 0.032). When patients were stratified based on primary site of the tongue and others, there was a significant difference in OS. This result may be due to the good OS of patients with the tongue as primary site. In the future, subgroup analysis of the primary site with a large sample size will be interesting.

**Table 5:** Studies evaluating the cutoff value of CAR in various cancers.

No	Authors	Year	Subjects (No. of patients)	Primary cancer	Significant CAR cutoff value
1	Yu et al	2018	160	Esophageal cancer	0.023
2	Liu et al	2015	455	Gastric cancer	0.025
3	Haruki et al	2016	113	Pancreatic cancer	0.030
4	Present study	2020	205	Oral cancer	0.032
5	Kinoshita et al	2015	186	Hepatocellular cancer	0.037
6	Park et al	2016	40	Oral cancer	0.085
7	Ishibashi et al	2018	143	Esophageal cancer	0.085
8	Wei et al	2015	423	Esophageal cancer	0.095
9	Kudou et al	2019	144	Esophagogastric junction and upper gastric cancer	0.100
10	Sun et al	2017	148	Nasopharyngeal cancer	0.189
11	Kuboki et al	2017	56	Hypopharyngeal and laryngeal cancer	0.320
12	Xu et al	2015	468	Esophageal cancer	0.500
13	Wang et al	2019	240	Oral cancer	0.525

In the subgroup analysis of the surgery group, OS was significantly associated with CAR, NLR, and SII. The cutoff value of CAR 0.032 derived from this study was a useful preoperative predictor for the surgery cohort. Although the recognized postoperative predictive risk factors for OSCC (ENE and positive LN counts ≥ 2) are more significant than CAR, CAR is also a meaningful factor as a preoperative predictor.

The levels of inflammatory components have a certain prognostic value in cancer^1^. In the present study, Cox multivariate regression analysis identified three independent predictive factors for OS: age, stage, and CAR. These results suggest that the prognosis of OSCC is defined not only by the clinical stage of the tumor and age, but also by systemic host factors. Therefore, a combination analysis with age, clinical stage as the tumor factor, and CAR as the host factor was performed. Significant differences in OS rates were observed between groups when the patients were subdivided according to CAR, age, and tumor stage. The most favorable survival outcomes were obtained for those with a low CAR value (< 0.032), low stage (I or II), and low age (< 71.3), while the opposite was true for those presenting with a high CAR value (≥ 0.032), high stage (III or IV), and high age (≥ 71.3). Although a high CAR value (≥ 0.032), tumor stage I or II, and low age (< 71.3) had the worst survival outcomes, the case count was only 4 and the significance was unknown. In a study that investigated the predictive utility of CAR in esophageal cancer, patients with a high CAR usually experienced severe tumor-related inflammatory reactions or poor nutritional status; these patients may benefit from anti-inflammatory therapy or nutritional support. Anti-inflammatory therapy and nutritional support should be added to the individualized treatment regimen of patients with a high CAR^6^. In OSCC, a high CAR was found to be associated with a less favorable prognosis. Likewise, those presenting with a high CAR and stage III or IV cancer may require a more aggressive treatment regimen, including radiotherapy and chemotherapy, than indicated by their general condition. Additional long-term studies will be necessary to validate this finding.

As CAR can be readily assessed through routine blood tests, it is a useful, simple, objective, reproducible, and economically feasible prognostic indicator in patients with OSCC. Limitations of the present study include its retrospective, single-institute design and possible selection bias during patient and data collection. In addition, there was considerable heterogeneity in the treatment provided to the patients. Therefore, a long-term prospective multicenter study will be required to validate the preliminary findings of the present report.

In conclusion, among inflammation-based prognostic markers, the AUC was highest for CAR, and in the Cox multivariate analysis, CAR (≥ 0.032 vs. < 0.032), in addition to age and stage, was identified as an independent predictor and, thus, a useful prognostic marker in OSCC. CAR is a novel inflammation-based prognostic marker for patients with OSCC.

## Materials and methods

### Study design and patients

The present retrospective cohort study included patients diagnosed with OSCC who underwent treatment between 2013 and 2018 at the Department of Oral and Maxillofacial Surgery, University of Tsukuba Hospital, Ibaraki, Japan. From an initial sample size of 259, 54 patients were excluded because they did not undergo treatment and/or palliative therapy. Hence, a total of 205 patients were included in this study. Cancer was staged according to the 2017 Union for International Cancer Control categories (8th edition). The main initial treatments were surgery for patients with resectable tumors in an operable general condition. According to the postoperative pathological results, the high-risk group (ENE +, LN counts ≥ 2, close or positive margin) was treated with adjuvant radiotherapy (60–66 Gy) and/or chemotherapy (CDDP 100 mg/m^2^, 2 or 3 courses). Salvage therapy was mainly radiotherapy (70 Gy) with chemotherapy (CDDP 100 mg/m^2^, 2 or 3 courses). The follow-up duration was every 2 to 4 weeks in the first year, 2 months in 2 years, 3 months in 3 years, 4 months in 4 years, and 5 months in 5 years with CT and MRI examination. This study was conducted in accordance with the Declaration of Helsinki and was approved by the Institutional Review Board of the University of Tsukuba Hospital. Informed consent was waived due to the retrospective nature of the study (No. R02-117).

### Study variables

The primary predictor variables were inflammation-based markers. The ROC curve, AUC, sensitivity, specificity, and 95% CI were calculated to determine the best-defined risk groups as follows: preoperative blood examination data (NLR, SIRI, SII, LMR, PLR, CAR). The AUC was measured to evaluate and compare the discrimination ability of the variables. The patients were divided into binary subgroups using the best-defined preoperative blood examination data (NLR, SIRI, SII, LMR, PLR, and CAR) as the cutoff point. The cutoff values for predicting OS were determined by ROC curve analysis based on the maximum Youden index. The primary outcome variable was OS, and the other variables were related to patient characteristics, including sex, age, and tumor stage.

### Statistical analyses

CAR was selected as the primary predictor variable, as the highest AUC value was calculated for this inflammatory marker relative to the other inflammation-based markers that were examined. The optimal cutoff level for CAR was 0.032 for OS. Patients were divided into high CAR (≥ 0.032) or low CAR (< 0.032) subgroups using this cutoff value, and differences between the subgroups were analyzed for significance. Survival curves were plotted according to the Kaplan–Meier method, and any differences were analyzed using the log-rank test. OS was calculated from the date of first diagnosis to death from any cause. The cutoff date for surviving patients was May 2020. Subgroups were compared using the Mann–Whitney U test and Chi-square test. Univariate and multivariate analyses for OS were performed using a Cox proportional hazards model. Logistic multivariate analysis of the parameters with stepwise forward selection method was used to identify independent variables for multivariate Cox regression analysis. All statistical analyses were performed using the Statistical Package for the Social Sciences (SPSS) software version 25 for Macintosh (SPSS, Chicago IL, USA). *P* < 0.05 was considered statistically significant.

## Supplementary Information

Supplementary Table.

Supplementary Figure.
