# Supplementary material for: Association between the C-reactive protein/albumin ratio and prognosis in patients with oral squamous cell carcinoma
Source: Sci Rep. 2021 Mar 8;11:5446. doi: 10.1038/s41598-021-83362-2 (PMC7940640; doi:10.1038/s41598-021-83362-2)
Supplement: Supplementary file 1 — Supplementary Table. [file 41598_2021_83362_MOESM1_ESM.docx]

﻿﻿**Association between the C-reactive protein/albumin ratio and prognosis in patients with oral squamous cell carcinoma**

**Kenji Yamagata *, Satoshi Fukuzawa, Naomi Ishibashi-Kanno, Fumihiko Uchida, Hiroki Bukawa**

**Supplementary Table 1** Logistic multivariate analysis for OS with stepwise forward selection method

| Variables | B | OR (95% CI) | *P* values ^†^ |
| --- | --- | --- | --- |
| Age |  |  |  |
| ≥71.3 vs.<71.3 | -0.763 | 0.466(0.235-0.924) | 0.029* |
| Stage |  |  |  |
| I, II vs. III, IV | -1.312 | 0.269(0.125-0.578) | 0.001** |
| CAR |  |  |  |
| ≥0.032 vs. <0.032 | -1.115 | 0.328(0.166-0.648) | 0.001** |

† By stepwise forward selection method.

* *P* < 0.05 Statistically significant difference, ***P* < 0.01 Statistically significant difference

OR, Odds ration; CAR, C-reactive protein/albumin ratio

**Figure Legend**

**Supplementary Figure 1.** Kaplan–Meier survival curve according to clinical staging. There were significant differences in OS when the patient cohort was stratified according to clinical stage (OS rates: stage I, 88.0%; stage II, 71.5%; stage III, 77.4 %; stage IVA, 49.4%; stage IVB, 44.9% and stage IVC, 0%; *P*<0.001).
