# Supplementary figures and images for: Association between the C-reactive protein/albumin ratio and prognosis in patients with oral squamous cell carcinoma
Source: Sci Rep. 2021 Mar 8;11:5446. doi: 10.1038/s41598-021-83362-2 (PMC7940640; doi:10.1038/s41598-021-83362-2)

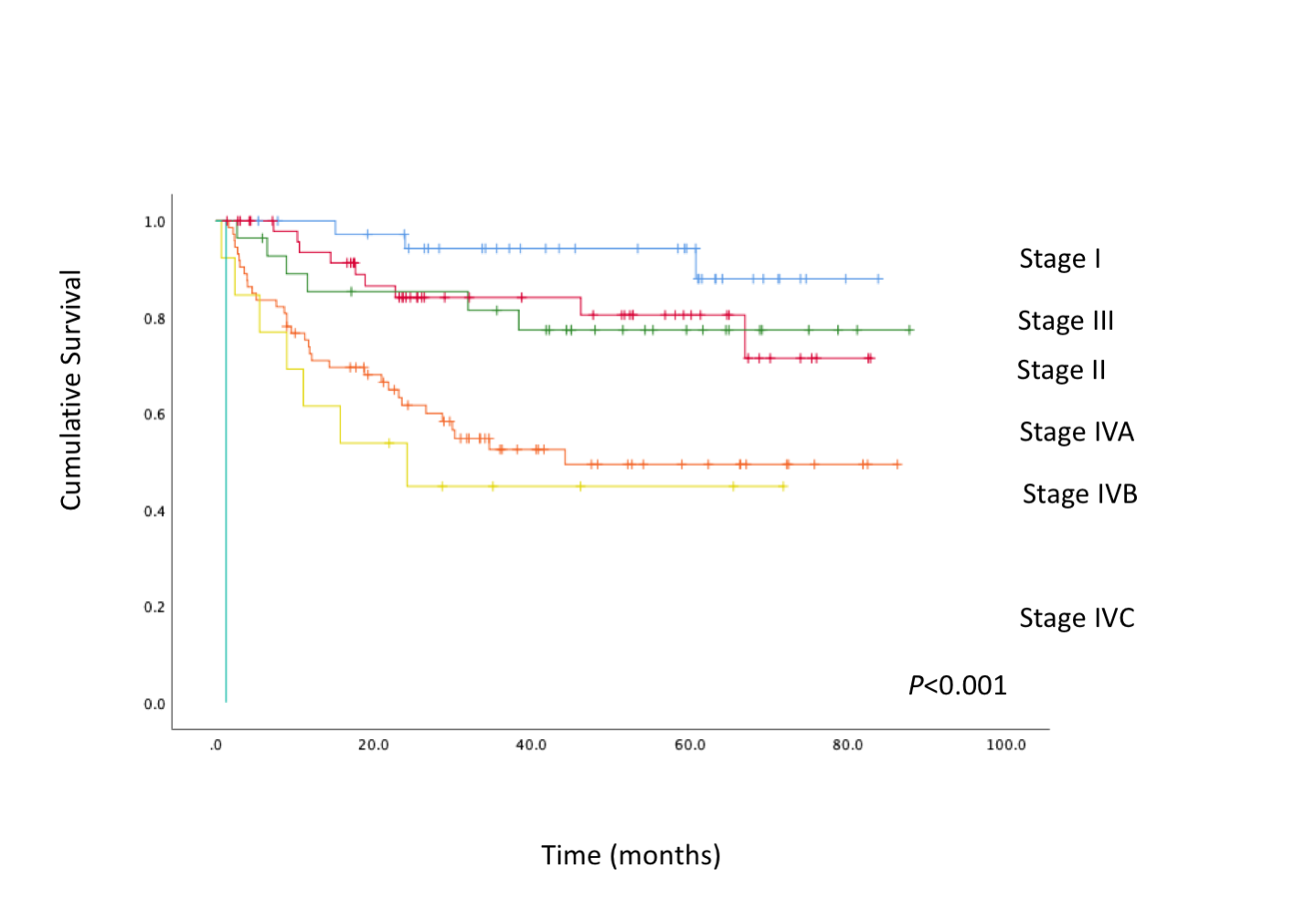

Supplement: Supplementary file 2 — Supplementary Figure. [file 41598_2021_83362_MOESM2_ESM.tiff]
